# Altered gut microbial profile is associated with differentially expressed fecal microRNAs in patients with functional constipation

**DOI:** 10.3389/fmicb.2023.1323877

**Published:** 2024-01-11

**Authors:** Junpeng Yao, Xiangyun Yan, Yanqiu Li, Yaoyao Chen, Xianjun Xiao, Siyuan Zhou, Wei Zhang, Lu Wang, Min Chen, Fang Zeng, Ying Li

**Affiliations:** ^1^Acupuncture and Tuina School, Chengdu University of Traditional Chinese Medicine, Chengdu, Sichuan, China; ^2^School of Health Preservation and Rehabilitation, Chengdu University of Traditional Chinese Medicine, Chengdu, Sichuan, China; ^3^Teaching Affairs Office, Chengdu University of Traditional Chinese Medicine, Chengdu, Sichuan, China; ^4^Department of Colorectal Disease, Hospital of Chengdu University of Traditional Chinese Medicine, Chengdu, China

**Keywords:** functional constipation, gut microbiota, microRNA, 16S rRNA, microRNA sequencing

## Abstract

While dysbiosis within the intestinal ecosystem has been associated with functional constipation (FC), the mechanisms underlying the interactions between FC and the microbiome remain poorly elucidated. Recent investigations suggested that host microRNAs (miRNAs) can modulate bacterial growth and influence the composition of the gut microbiome. To explore the connection between gut microbiota and fecal miRNAs in FC patients, we initially employed 16S rRNA sequencing to assess the gut microbial landscape in 30 FC patients and 30 healthy controls (HCs). The α-diversity within the FC group exhibited some alterations, and the β-diversity significantly differed, signifying distinctive variations in gut microbiota composition between FC patients and HCs. Subsequently, we identified 44 differentially expressed (DE) miRNAs in feces from FC patients and HCs. Through correlation analysis between DE miRNAs and FC-associated microbiota, we detected an interaction involving nine DE miRNAs (*miR-205-5p*, *miR-493-5p*, *miR-215-5p*, *miR-184*, *miR-378c*, *miR-335-5p*, *miR-514a-3p*, *miR-141-3p*, and *miR-34c-5p*) with seven bacterial genera (*Oscillibacter*, *Escherichia.Shigella*, *UCG.002*, *Lachnospiraceae_NK4A136_group*, *Lachnospiraceae_UCG.010*, *Eubacterium_ruminantium_group* and *Megamonas*), as evidenced by a co-occurrence network. Further, a comprehensive panel of seven diagnostic biomarkers (*Oscillibacter*, *Escherichia.Shigella*, *UCG.002*, *miR-205-5p*, *miR-493-5p*, *miR-215-5p*, and *Lachnospiraceae_NK4A136_group*) demonstrated robust discriminatory capacity in predicting FC status when integrated into a random forest model (AUC = 0.832, 95% CI: 65.73–98.88). Microbiomes correlating with DE miRNAs exhibited enrichment in distinct predicted metabolic categories. Moreover, miRNAs correlated with FC-associated bacteria were found to be enriched in signaling pathways linked to colonic contractility, including Axon guidance, PI3K-Akt signaling pathway, MAPK signaling pathway, and Hippo signaling pathway. Our study offers a comprehensive insight into the global relationship between microbiota and fecal miRNAs in the context of FC, presenting potential targets for further experimental validation and therapeutic interventions.

## Graphical Abstract

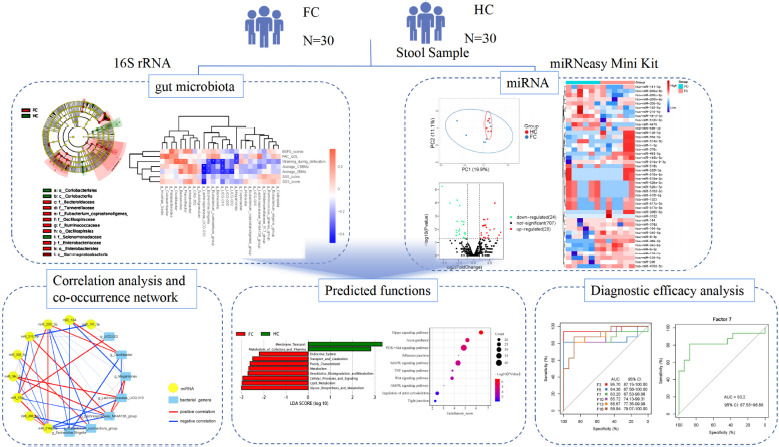

## Highlights

• This study suggested functional constipation (FC) patients display distinct microbial profiles and identified 26 potential FC-associated bacteria.
• This study identified unique fecal miRNA patterns in FC patients.
• Correlations were found between the abundance of seven bacterial genera and nine fecal miRNAs.
• This study might shed light on a novel underlying mechanism by which miRNAs can selectively recruit microbes by orchestrating glycan biosynthesis and enhancing axon guidance in the enteric nervous system.

## 1 Introduction

Functional constipation (FC) is characterized by chronic and non-organic causes of constipation, excluding cases associated with irritable bowel syndrome, structural abnormalities, or metabolic disorders (Black et al., 2020). FC is a prevalent functional bowel disorder on a global scale, with an incidence of approximately 17.5% in the Americas and the Asia Pacific region, 8.75% in Europe, and ranging from 3 to 17% in China (Palsson et al., 2020). While FC does not pose an immediate threat to life, its substantial disease burden cannot be underestimated. Prolonged constipation may contribute to the development of colon cancer, and straining during defecation can increase the risk of cardiovascular and cerebrovascular events in affected individuals (Power et al., 2013; Dong et al., 2023). Moreover, certain metabolites produced by gut bacteria can influence brain function (Morais et al., 2021). As the precise pathophysiological mechanisms underlying FC remain incompletely understood, current treatment approaches primarily focus on symptom management. However, long-term reliance on laxatives can lead to adverse reactions such as laxative dependence, melanosis coli, and electrolyte imbalances (Bharucha and Lacy, 2020; Shah et al., 2021; Sharma et al., 2021). Therefore, investigating the pathogenesis of FC and enhancing treatment efficacy hold substantial clinical significance.

The intestinal microbial ecosystem represents the largest and most vital microbial ecosystem of the organism, playing a pivotal role in activating and sustaining intestinal physiological functions (Jia et al., 2018; Fan and Pedersen, 2021). Research by Parthasarathy et al. (2016) has revealed alterations in the gut microbiota composition of patients with constipation. Notable changes include significant reductions in the populations of beneficial bacteria such as *Lactobacillus* and *Bifidobacterium*, alongside significant increases in the abundance of pathogenic bacteria like *Enterobacter* and *Escherichia coli* (Parthasarathy et al., 2016). Functional studies have demonstrated that the gut microbiota of patients with FC can modulate the levels of 5-hydroxytryptamine (5-HT) in the intestinal tract by influencing the expression of tryptophan hydroxylase-2 (TPH2). This modulation, in turn, impacts local neural activity within the intestinal tract, regulating intestinal secretion and motility (Bhattarai et al., 2018). Additionally, there is a significant increase in bacteria involved in the metabolism of butyric acid and bile acids within the gut microbiota of FC patients (Fan et al., 2022). These findings collectively underscore an intricate interplay between gut microorganisms and the host in individuals with constipation, although the precise key regulatory elements governing this interaction remain elusive.

MicroRNAs (miRNAs), which have been highly conserved throughout evolution, represent a class of endogenous, non-coding single-stranded RNAs approximately 22 nucleotides in length (Yao et al., 2022). They exert a post-transcriptional level of control by negatively regulating the expression of target genes through complementary binding with specific sequences on mRNAs (Hong et al., 2021). Mazzone et al. (2020) identified 13 significantly up-regulated miRNAs in the colon mucosa of FC patients. Additionally, in animal experiments, miR-129 was observed to be markedly overexpressed in colon tissues in an FC mouse model, and it was found to influence the distribution and function of Interstitial Cells of Cajal (ICC) through negative regulation of the N-acetylgalactosaminyltransferase-like 1/transforming growth factor-β1 signaling pathway (Wang et al., 2022). All these studies suggested that miRNAs are involved in the pathophysiology of FC and have the potential to be a non-invasive marker for the diagnosis and assessment of FC. Recent findings by Liu et al. (2016) further revealed that miRNAs in intestinal epithelial cells and Hopx-positive cells could enter the gut microbiota to modulate and regulate the transcription of microbiota-related genes, providing insight into how host cells can regulate microbial communities. However, our understanding of the role of miRNAs in the interplay between the host and gut microbiota in the context of FC remains limited.

To deepen our comprehension of the gut microbiota structure in FC and elucidate the relationship between gut microbiota and fecal miRNAs, this study first employed 16S rDNA sequencing and small RNA sequencing techniques to analyze the gut microbiota structure and fecal miRNA expression profiles. Subsequently, correlation analyses were conducted between the identified miRNAs and gut microbiota, and a random forest model was constructed to assess the collective diagnostic potential of these biomarkers. Finally, pathway analysis was employed to predict the biological functions of these miRNAs and gut microbiota. To the best of our knowledge, this study represents the first attempt to investigate the associations between miRNA expression and the microbiome within the context of FC.

## 2 Results

### 2.1 Information of cohort FC patients and healthy subjects

A total of 30 subjects with a clinical diagnosis of FC (age, 30.00 [8.50]; sex, male: female, 3:27) and 30 healthy individuals (age, 29.50 [18.00]; sex, male: female, 4:26) were recruited from February of 2022 to December of 2022 (Table 1). There was no statistical difference in age, gender, and body mass index between the two groups (*p*-value > 0.05), indicating these characteristics are matched. Defecation frequency and BSFS scores were statistically decreased (*p*-value < 0.01), while the defecation difficulties and PAC-QOL scores were significantly increased in FC patients when compared to the healthy individuals (*p*-value < 0.01).

**TABLE 1:** Demographics and characteristics of study cohort.

	Healthy subjects	FC patients	*p*-value
Subjects (n)	30	30	N/A
Age (years)	30.00 [8.50]	29.50 [18.00]	0.50
Sex, male/female	3/27	4/26	0.50
Body mass index (kg/m^2^)	21.51 ± 2.09	20.94 ± 2.49	0.34
Disease duration (months)	N/A	121.00 [111.75]	N/A
Average of CSBMs per week (n)	7.00 [0.00]	1.50 [3.00]	<0.001
Average of SBMs per week (n)	7.00 [1.00]	2.00 [2.25]	<0.001
Straining during defecation	0.00 [0.19]	1.00 [0.08]	<0.001
BSFS scores	4.09 ± 0.40	2.91 ± 1.14	<0.001
PAC-QOL scale	1.13 ± 0.09	2.50 ± 0.62	<0.001
Self-rating depression scale	34.92 ± 8.08	40.38 ± 9.02	0.292
Self-rating anxiety scale	34.38 [11.25]	38.75 [12.50]	0.004

FC, functional constipation; CSBMs, complete spontaneous bowel movements; SBMs, spontaneous bowel movements; BSFS, Bristol stool form scale; PAC-QOL, patient-assessment of constipation quality of life.

### 2.2 Alterations of gut microbiota composition in FC patients based on 16S rRNA data

**FIGURE 1:**
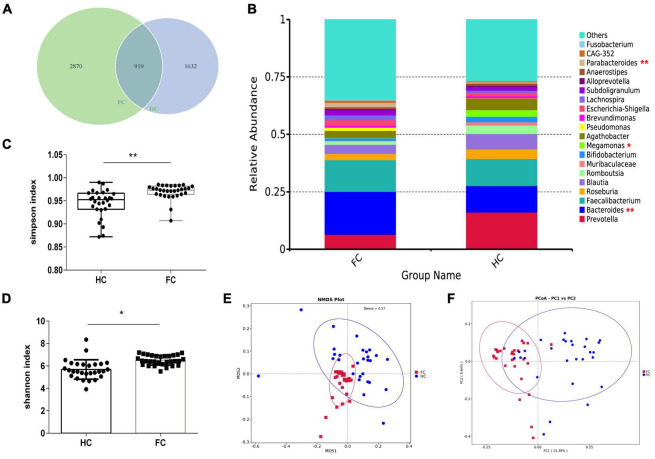
The shift of gut microbiota in FC patients and HCs based on the 16S rDNA data. **(A)** Venn diagram of the observed ASVs in FC and HC. **(B)** The relative abundance between two groups at the level of genus (Top 20). **(C,D)** Difference analysis of Shannon and Simpson index between the FC and HC groups. **(E,F)** PCoA and NMDS of the microbiota based on the unweighted UniFrac distance metrics. ANOSIM, *R* = 0.3742, *p* = 0.004. *p < 0.05 and **p < 0.01 are indicated.

The rarefaction curves and species accumulation boxplots were generated from ASVs, with 97% identity achieved in all samples (Supplementary Figure 1). This indicated that the testing samples were sufficient and the amount of data were reasonable for the investigation of fecal microbiota. The FC group and the healthy control (HC) group displayed 2,870 and 1,632 unique ASVs, respectively. A total of 919 ASVs were shared by both groups (Figure 1A). Taking the genus level as an example, there was a difference in the relative abundance of species between the FC group and the HC group. *Bacteroides* and *Parabacteroides* were significantly increased, while *Megamonas* were decreased in the FC group (*p*-value < 0.01 or 0.05) (Figure 1B). The results of seven indexes in α-diversity analysis are shown in Table 2, Supplementary Figure 2, and Figures 1C, D. It is indicated that the α-diversity of gut microbiota in FC patients was richer than HCs, there were significant differences in the 6 indexes (*p*-value < 0.01 or 0.05), except for the goods coverage index. The PCoA and NMDS were performed to investigate the extent of the similarity of the microbial communities in the two cohorts based on unweighted UniFrac distance metrics (Figures 1E, F). These analyses indicated that the microbiota composition of the FC group clusters was more heterogeneous and significantly different from that of the HC group.

**TABLE 2:** α-diversity indices comparing FC patients to healthy subjects.

α -diversity index	Healthy subjects	FC patients	*p*-value
Chao1	278.88 [110.40]	366.17 [146.17]	<0.001
Dominance	0.05 [0.04]	0.03 [0.02]	<0.001
Goods coverage	1.00 ± 0.00	1.00 [0.00]	0.309
Observed otus	278.50 [110.00]	364.00 [142.00]	<0.001
Pielou_e	0.69 ± 0.07	0.76 ± 0.04	0.022
Shannon	5.68 ± 0.87	6.51 ± 0.44	0.030
Simpson	0.95 [0.04]	0.97 [0.02]	<0.001

FC, functional constipation. Pielou_e and Shannon index were tested by *t*-test; Chao1, Dominance, Goods coverage, Observed_otus and Simpson index were tested by *Wilcoxon Mann-Whitney U* test.

**FIGURE 2:**
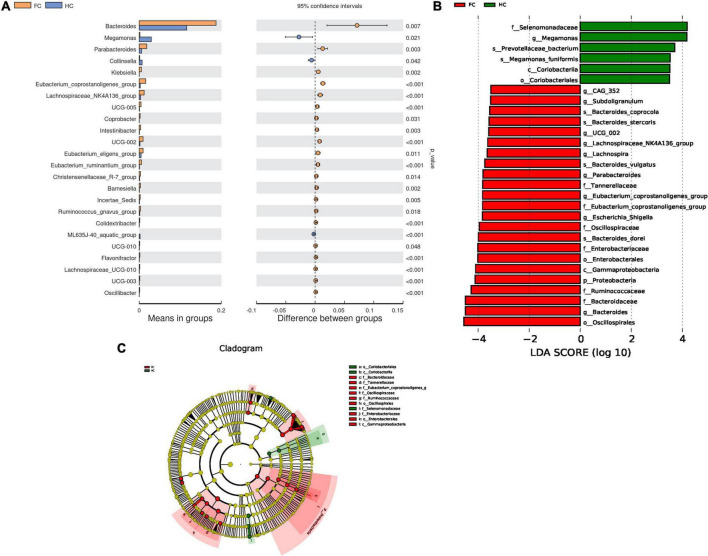
Differential microbes in FC patients and HCs. **(A)** The bar plots of the relative abundance of differential genera based on *t-*test. **(B)** LDA scores for the bacterial taxa are differentially abundant between groups (LDA > 3.5). Green bars indicate taxa with enrichment in FC, and red bars indicate taxa with enrichment in HC. **(C)** Evolutionary cladograms generated by LEfSe indicate differences in the bacterial taxa between groups. Green bars indicate taxa with enrichment in FC, and red bars indicate taxa with enrichment in HC.

To identify microbes with significantly different relative abundance in the two cohorts, a *t*-test was carried out (Figure 2A). As compared with HCs, the abundance of 22 genera, including *Bacteroides* (*p* = 0.007) and *Parabacteroides* (*p* = 0.003) were significantly increased, while the abundance of *Megamonas* (*p* = 0.021) and *Collinsella* (*p* = 0.042) were decreased in FC patients (Figure 2A). Meanwhile, LEfSe analysis identified 29 significantly different microbes between the two groups, the LDA distribution diagram analysis (LDA > 3.5) showed a clear alteration of the microbiota characterized by higher *CAG_352*, *Subdoligranulum*, *UCG_002*, *Lachnospira*, *Parabacteroides*, *Lachnospiraceae_NK4A136_group*, *Eubacterium_coprostanoligenes_group*, *Escherichia_Shigella*, and *Bacteroides* levels in FC group (Figures 2B, C). However, *Megamonas* levels were significantly decreased in FC patients (Figures 2B, C).

### 2.3 Sequencing analysis revealed differentially expressed miRNAs between the two groups

**FIGURE 3:**
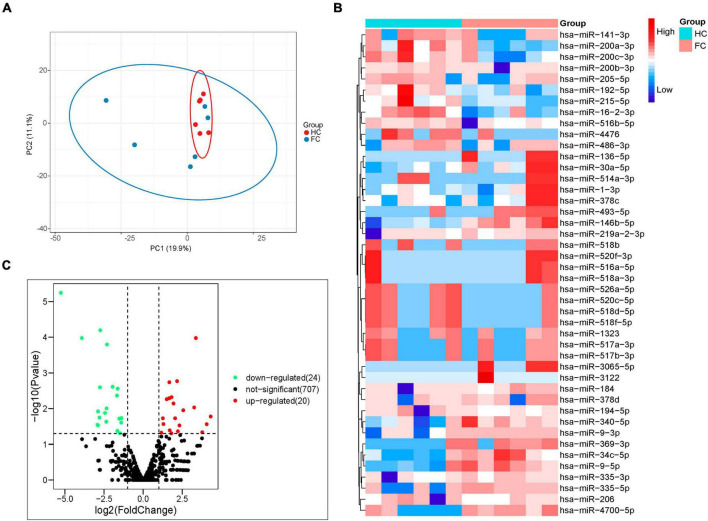
Analysis of fecal miRNAs in FC patients and HCs by the next generation RNA sequencing. **(A)** PCA of differential miRNAs distinguishes two cohorts. **(B)** Volcano plot showing differential expression of miRNAs. Differential miRNAs expression is shown by red (upregulated) versus blue (downregulated) intensity. The Y-axis depicts a 2.0-fold up or down change, while the X-axis represents a *p*-value of 0.05. **(C)** Heatmap of differentially expressed miRNAs; 20 upregulated and 24 downregulated miRNAs are presented.

Through miRNA high-throughput sequencing analysis, 1,844 miRNAs were obtained from all samples, including 1,047 known miRNAs and 797 newly predicted miRNAs. After alignment of miRNA sequence data against the human miRNA database (miRBase v21),^1^ principal component analysis (PCA) visualized the sample distribution in a two-dimensional scatter plot, the contribution of PC1 and PC2 to the overall variance in the data set is 19.9 and 11.1%, respectively (Figure 3A). These data indicated that the overall miRNA expression pattern was distinguished according to the presence of FC.

The fecal miRNAs differentially expressed between patients with FC and HCs were determined according to a statistical criterion of *p-value* < 0.05, log2FoldChange (log2FC) ≥ 1 and CPM ≥ 1. A total of 44 significantly different miRNAs were identified, with 20 upregulated and 24 downregulated in FC group compared to HC group (Figure 3B). The heatmap of the 44 different miRNAs of the FC and HC groups is shown in Figure 3C.

### 2.4 The preliminary screening results of the differential expressed miRNAs and constipated-associated gut microbiota

In terms of sequencing results, miRNAs with extremely low signals (base mean = 0) were not selected. As shown in Tables 3, 4, the top 10 differentially expressed (DE) miRNAs with higher enrichment in FC patients include *miR-4700-5p*, *miR-493-5p*, *miR-514a-3p*, *miR-369-3p*, *miR-335-5p*, *miR-34c-5p*, *miR-378d*, *miR-335-3p*, *miR-378c*, and *miR-184* (Table 3); meanwhile, *miR-4476*, *miR-215-5p*, *miR-526a-5p*, *miR-520c-5p*, *miR-518*, *miR-141-3p*, *miR-205-5p*, and *miR-517* were observed at higher enrichment in healthy controls (Table 4).

**TABLE 3:** Top 10 upregulated fecal miRNAs in FC patients compare with HCs.

Mature_ID	baseMean_HC	baseMean_FC	log2FoldChange	*p*-value
hsa-miR-4700-5p	71.815615350	693.827634690	3.366860705	0.000105565
hsa-miR-493-5p	16.704200050	173.056229683	3.258242335	0.009301524
hsa-miR-514a-3p	49.673742633	253.023384167	2.560963284	0.011150233
hsa-miR-369-3p	26.175798417	188.508430743	2.31652355	0.029673918
hsa-miR-335-5p	55.940851755	269.550745500	2.219740171	0.043330153
hsa-miR-34c-5p	214.96979333	1041.70131158	2.16592952	0.001705622
hsa-miR-378d	118.96303679	446.120967775	2.041749259	0.018700679
hsa-miR-335-3p	177.38402455	733.078402583	1.939176065	0.007201004
hsa-miR-378c	431.04086138	1420.23373135	1.814298629	0.004796602
hsa-miR-184	85.769245733	297.110001637	1.802566638	0.049538533

**TABLE 4:** Top 10 downregulated fecal miRNAs in FC patients compare with HCs.

Mature_ID	baseMean_HC	baseMean_FC	log2FoldChange	*p*-value
hsa-miR-4476	704.389441	22.493451	5.26066761917007	0.000000569
hsa-miR-215-5p	16319.510613	1085.697440	3.92030432655696	0.000105701
hsa-miR-526a-5p	101.571103228	4.142552805	2.92188708907852	0.02845614
hsa-miR-520c-5p	101.571103228	4.142552805	2.92188708907852	0.02845614
hsa-miR-518f-5p	101.571103228	4.142552805	2.92188708907852	0.02845614
hsa-miR-518d-5p	101.571103228	4.142552805	2.92188708907852	0.02845614
hsa-miR-141-3p	316.647900822	42.55817459	2.7580489273887	0.002529715
hsa-miR-205-5p	853.539032167	98.04627931	2.7422651145099	0.0000063907
hsa-miR-517b-3p	231.263391800	25.04197379	2.44023464326948	0.013306236
hsa-miR-517a-3p	231.263391800	25.04197379	2.44023464326948	0.013306236

**FIGURE 4:**
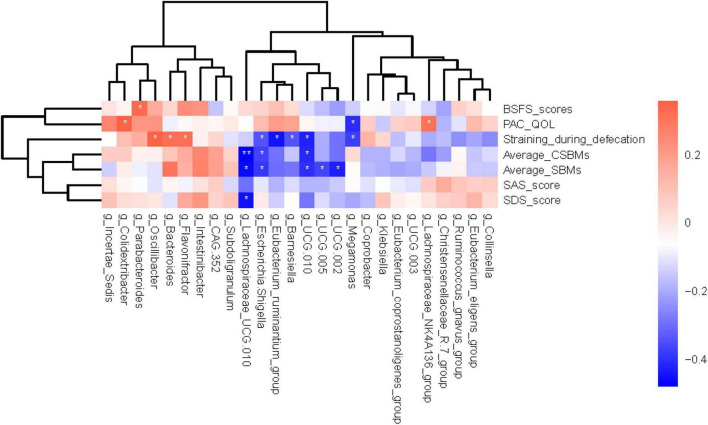
Heatmap showing bacterial genera that were significantly correlated with the constipated-indicators. Blue indicates negative correlations, and red indicates positive correlations. The color depth was related to the absolute value of the correlation coefficient. *FDR-corrected *p* < 0.05 and **FDR-corrected *p* < 0.01 are indicated.

Correlation analysis can help measure the closeness of significant gut microbiota to clinical indicators. With reference to the results of LEfSe analysis (Figures 2B, C) and relative abundance differences between groups (Figure 2A), 26 different gut genera were preliminary screened. The results of Spearman’s correlation analysis between these bacterial genera and constipated-manifestations are shown in Supplementary Table 2 and Figure 4, including *Lachnospiraceae_NK4A136_group*, *Megamonas*, *Lachnospiraceae_UCG.010*, *Escherichia.Shigella*, *Eubacterium_ruminantium_group*, *Barnesiella*, *UCG.010*, *UCG.005*, *UCG.002*, *Colidextribacter*, *Parabacteroides*, *Oscillibacter*, *Bacteroides* and *Flavonifractor* (*FDR*-corrected *p* < 0.05).

### 2.5 The correlation analysis and co-occurrence network construction among the DEMs and FC-associated microbiomes

**FIGURE 5:**
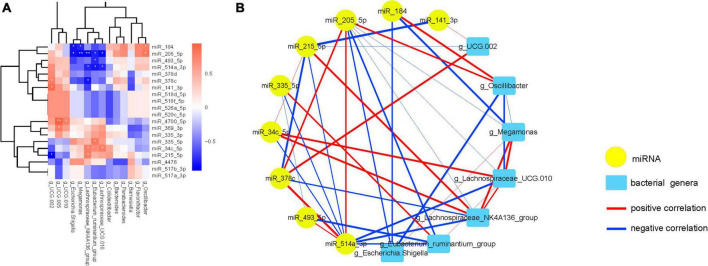
FC-associated microbiomes significantly correlated with most significantly DE miRNAs. **(A)** The heatmap shows the 20 most significantly DEMs correlated with FC-associated microbiomes. Positive correlations are shown in red, and negative correlations are shown in blue. **(B)** Interaction network showing the 11 DEMs and their correlated bacteria. Each node presents a miRNA or bacteria. Edge thickness represents the magnitude of the correlation, with red indicating positive correlation and with blue indicating negative correlation. *FDR-corrected *p* < 0.05 and **FDR-corrected *p* < 0.01 are indicated.

Subsequently, correlation analysis between the DE miRNAs and FC-associated gut microbiota was performed (Figure 5A; Supplementary Table 3). The correlations clearly show a distinct pattern based on the enrichment of miRNAs, 11 miRNAs that have statistically significant positive or negative correlations with 9 microflora (*FDR*-corrected *p* < 0.05).

Then, a co-occurrence network was constructed to visualize the relationships between these miRNAs and their correlated bacteria (Figure 5B; Supplementary Figure 3). The network showing the interconnection between these miRNAs and bacterial genera, which consists of 20 nodes and 43 edges with the average degree of connexions at 4.30 (The detailed information can be found in Supplementary Tables 4, 5).

Interestingly, *miR-205-5p* was inversely associated with *Escherichia.Shigella*, *Megamonas*, *Lachnospiraceae_NK4A136_ group*, *Eubacterium_ruminantium_group* and *Lachnospiraceae_ UCG.010*, while positively interacted with *miR-514a-3p*, *miR- 378c* and *Oscillibacter*. Furthermore, *miR-514a-3p* expression was negatively associated with *miR-335-5p*, *miR-34c-5p*, *miR- 215-5p*, *Lachnospiraceae_NK4A136_group*, *Eubacterium_ruminan tium_group* and *Lachnospiraceae_UCG.010*, and was positively associated with *miR-378c*. Additionally, an interaction among *miR-34c-5p*, *Eubacterium_ruminantium_group*, *miR-215-5p*, *Lachnospiraceae_NK4A136_group* and *Lachnospiraceae_UCG.010* was also observed. In the same way, *Escherichia.Shigella* abundance was negative related to *miR-184* and *Oscillibacter*, and was positive related to the abundance of *Megamonas*. Experimental validations are required to investigate these correlations.

Moreover, the expression of *miR-4700-5p* was positive related to *UCG.010* and *UCG.005*, and the expression of *miR-369-3p* was positive related to the abundance of *UCG.005* (Supplementary Figure 3).

### 2.6 Predicted functions of microbiomes correlated with DE miRNAs

**FIGURE 6:**
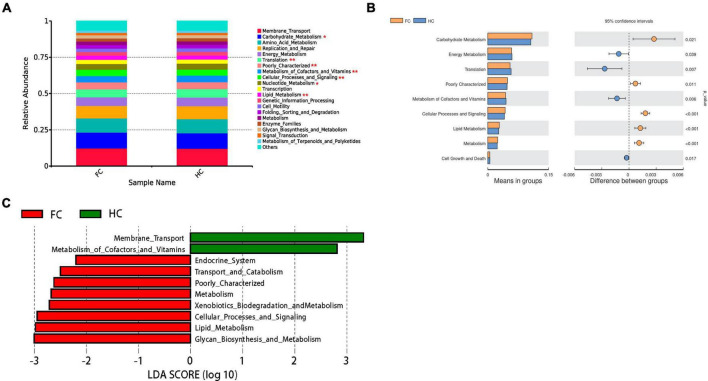
PICRUSt analyses of the microbiomes correlated with DE miRNAs. **(A)** The average abundance of KEGG pathway differentially enriched in HC and FC according to level 2. **(B)** The bar plots of the relative functional abundance between groups based on *Wilcoxon rank-sum* test. **(C)** LDA scores for the KEGG functions showed different abundances between HC and FC. Only taxa with LDA > 2.0 are shown.

To confirm the findings of the analysis of the interaction between certain gut microbiota and miRNAs, bioinformatics predictions were conducted. The KEGG information corresponding to the ASVs was obtained using PICRUSt software (Figure 6). Meanwhile, all the KEGG pathways based on level 2 were disrupted in FC, relative to HC group (Figure 6A). There were significant differences in carbohydrate metabolism (*p* = 0.021), energy metabolism (*p* = 0.039), translation (*p* = 0.007), metabolism of cofactors and vitamins (*p* = 0.006), cellular processes and signaling (*p* < 0.001), lipid metabolism (*p* < 0.001) and cell growth and death (*p* = 0.017) between two groups (Figure 6B). The LEfSe analysis results based on the prediction of KEGG pathway are shown in Figure 6C. The differential microbiomes in the FC group were closely related to glycan biosynthesis and metabolism, lipid metabolism, cellular processes and signaling, xenobiotics biodegradation and metabolism, metabolism, transport and catabolism, and endocrine system (Figure 6C).

### 2.7 Predicted functions of miRNAs correlated with FC-associated microbiomes

**FIGURE 7:**
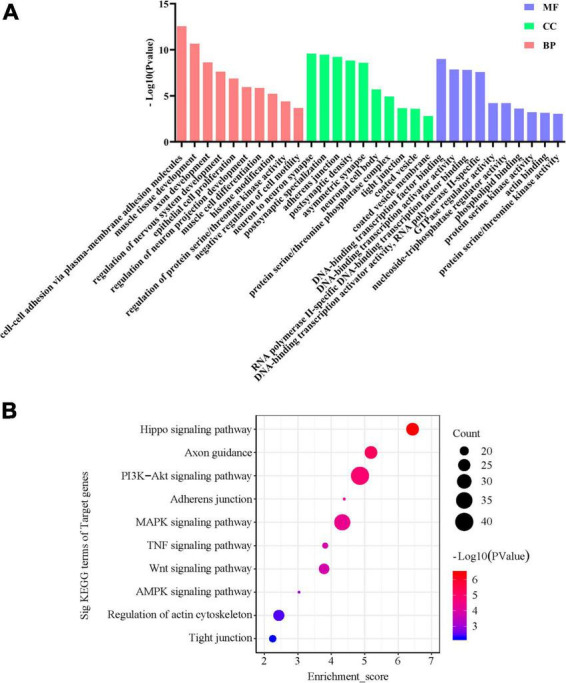
GO and KEGG signaling pathway analyses of the differentially expressed miRNAs correlated with FC-associated microbiomes. **(A)** Gene Ontology designations and **(B)** KEGG pathways were generated via DAVID database. Only terms with −Log_10_ *p*-value > 2 are presented.

Then, we investigate the function of miRNAs correlated with FC-associated microbiomes. We hypothesized that if these bacteria affect FC through modulating miRNA expression, then miRNAs that are significantly correlated with the bacteria should show enrichment in constipation-related genes and pathways. The target genes of the miRNAs can be found in Supplementary Figure 4 and Supplementary Table 6, while the MF, CC, and BP analysis of the target genes of the miRNAs are shown in Figure 7A. The top 5 statistically significantly enriched BP terms were Axon development (-Log_10_ *p*-value = 9.628), Muscle tissue development (-Log_10_ *p*-value = 11.67), Regulation of nervous system development (-Log_10_ *p*-value = 8.63), Epithelial cell proliferation (-Log_10_ *p*-value = 7.88), and Cell-cell adhesion via plasma-membrane adhesion molecules (-Log_10_ *p*-value = 13.57). Then, we predicted the functions of miRNAs by assigning pathways to the miRNA targets using the KEGG database. As shown in Figure 7B, the top 5 significantly enriched KEGG pathways were the PI3K-Akt signaling pathway (-Log_10_ *p*-value = 4.86), MAPK signaling pathway (-Log_10_ *p*-value = 4.33), Hippo signaling pathway (-Log_10_ *p*-value = 6.43), Axon guidance (-Log_10_ *p*-value = 5.19), and Regulation of actin cytoskeleton (-Log_10_ *p*-value = 2.43). Overall, the results of the GO and KEGG analyses indicate that fecal miRNAs correlated with FC-associated bacteria are involved in the regulation of colonic smooth muscle contractility and motility in FC patients.

### 2.8 Diagnostic efficacy analysis of associated DE miRNAs and microbiota in patients with FC

**FIGURE 8:**
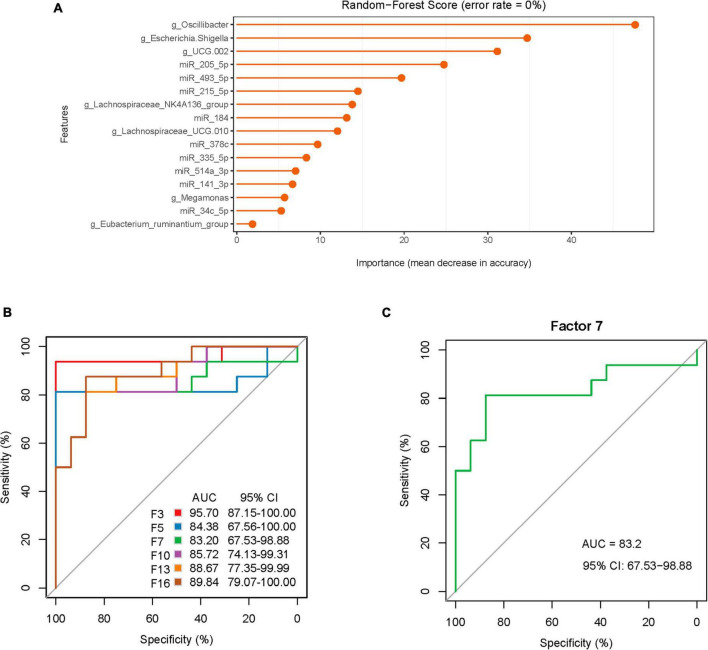
Random forest model prediction of diagnostic efficacy of associated DE miRNAs and microbiota between FC patients and healthy controls. **(A)** The importance of associated features from the model with the highest accuracy. **(B)** The random forest model displaying the classification for FC and HCs employing 3, 5, 7, 10, 13, and 16 different genera or miRNAs. **(C)** The random forest model used 7 different genera or miRNAs.

To further explore the potential diagnostic biomarkers, the ROC curve and AUC index were drawn for statistical analysis. The random-forest model was applied to construct the diagnostic prediction model based on the correlation analysis results between the DE miRNAs and FC-associated microbiomes. The AUC and these indicators are shown in Table 5 and Figure 8A, which were regarded as potential diagnostic features. After comprehensive consideration of the AUCs and random-forest scores (Figures 8B, C), seven potential diagnostic biomarkers (including *Oscillibacter*, *Escherichia.Shigella*, *UCG.002*, *miR-205-5p*, *miR-493-5p*, *miR-215-5p*, and *Lachnospiraceae_NK4A136_group*) were screened out and showed a strong diagnostic ability to predict FC status with AUC = 0.832 (95% CI: 65.73–98.88). On the whole, combined use of these diagnostic features may accurately discriminate against FC patients and healthy populations.

**TABLE 5:** AUCs and importance of associated DE miRNAs and bacterial genera for the diagnosis of FC.

Features	AUCs	Random_forest score	*p*-value
*Oscillibacter*	0.99609375	47.60270751	0.0074308
*Escherichia.Shigella*	0.94140625	34.70327863	0.00000116
*UCG.002*	0.962890625	31.1279629	0.001357407
*miR-205-5p*	0.8203125	24.75648229	0.023738515
*miR-493-5p*	0.83203125	19.68565209	0.019342821
*miR-215-5p*	0.77734375	14.4763298	0.007411587
*Lachnospiraceae_NK4A136_group*	0.83203125	13.79572187	0.00000792
*miR-184*	0.798828125	13.14203929	0.001180187
*Lachnospiraceae_UCG.010*	0.8359375	12.04186135	0.001971689
*miR-378c*	0.734375	9.666136448	0.637159684
*miR-335-5p*	0.775390625	8.321658334	0.294937957
*miR-514a-3p*	0.6015625	7.031576587	0.037125322
*miR-141-3p*	0.734375	6.669248571	0.003933245
*Megamonas*	0.548828125	5.710350377	0.001074839
*miR-34c-5p*	0.7421875	5.304328568	0.020979493
*Eubacterium_ruminantium_group*	0.71484375	1.881095259	0.0000204

## 3 Discussion

This study employed a prospective cohort design, enrolling healthy individuals as controls and individuals diagnosed with FC as participants. Through 16S rDNA and miRNA sequencing, the initial investigation revealed substantial discrepancies in the composition of the gut microbiota between FC patients and the healthy controls. Specifically, *Oscillibacter*, *Escherichia.Shigella*, *UCG.002*, *Lachnospiraceae_NK4A136_group*, *Lachnospiraceae_UCG.010*, and *Eubacterium_ruminantium_group* exhibited elevated relative abundance in the FC patients compared to the healthy controls, while the relative abundance of *Megamonas* was notably reduced in the FC patients. Furthermore, we identified 44 DE miRNAs in the fecal samples when comparing FC patients with healthy subjects. Among these miRNAs, *miR-493-5p*, *miR-184*, *miR-378c*, *miR-335-5p*, *miR-514a-3p*, and *miR-34c-5p* were significantly up-regulated in the FC patients, while *miR-205-5p*, *miR-215-5p*, and *miR-141-3p* were significantly down-regulated. Moreover, network analysis unveiled interactions among the aforementioned DE miRNAs and seven dominant bacterial genera. The incorporation of these correlated miRNAs with bacterial genera in diagnosis models demonstrated robust diagnostic performance, introducing a novel approach for detecting FC through the lens of gut microbiota-miRNA interactions. Additionally, functional analysis revealed that miRNAs strongly associated with the gut microbiota were enriched in pathways related to axon guidance, hinting at a potential contribution of axon guidance mechanisms to the pathogenesis of FC.

The gut microbiota, constituting the largest microbial ecosystem within the human body, plays a pivotal role in numerous metabolic processes (Yan et al., 2022). Beyond its primary role in digestion and absorption, the gut microbiota serves various functions, including the promotion of intestinal motility, establishment of immune defenses, protection against pathogen infiltration and tumor development, and maintenance of overall growth, metabolism, and intestinal equilibrium (Pan et al., 2022; Xiao and Kashyap, 2022). Any perturbation or dysregulation of the microbial composition can lead to metabolic disruptions. Our findings demonstrated significant alterations in the structure, abundance, and co-occurrence patterns of gut microbiota in FC patients in comparison to healthy subjects, aligning with similar microbial shifts observed in prior research studies (Parthasarathy et al., 2016; Tigchelaar et al., 2016; Xie et al., 2021; Nikolaieva et al., 2022).

The biggest innovative finding of this study is that miRNAs likely mediate host-microbiome communication in the context of FC. Notably, an intricate interplay was observed involving *miR-205-5p*, the relative abundance of six distinct intestinal bacteria (*Oscillibacter*, *Escherichia.Shigella*, *Megamonas*, *Lachnospiraceae_NK4A136_group*, *Eubacterium_ ruminantium_group*, and *Lachnospiraceae_UCG.010*), and two specific miRNAs (*miR-514a-3p* and *miR-378c*). Chen et al. (2022b) previously demonstrated that *miR-205-5p* exhibited reduced expression in gastric cancer (GC) cells and its up-regulation exerted inhibitory effects on GC cell proliferation. *Escherichia.Shigella*, a relatively uncommon pathogenic bacterium within the human microbiota and a member of the Enterobacteriaceae family, can modulate the onset and progression of constipation by producing docosapentaenoic acid, which in turn reduces gastrointestinal motility (Chen et al., 2022a). *Oscillibacter*, *Lachnospiraceae_NK4A136_group*, and *Lachnospiraceae_UCG.010* fall under the category of short-chain fatty acids (SCFAs) producing bacteria (Zhang X. et al., 2023). The SCFAs have a direct impact on enterocytes, prompting the expression of TPH1. This, in turn, promotes the synthesis of colonic 5-HT, thereby enhancing intestinal motility (Reigstad et al., 2015). *Eubacterium_ruminantium_group*, on the other hand, participates in downstream bacterial lipopolysaccharide metabolic pathways. It interacts with the toll-like receptor 4 and activates the molecule myeloid differentiation factor 88/nuclear factor-κB pathway in immune cells, ultimately leading to the secretion of pro-inflammatory cytokines such as interleukin-6, interleukin-1, and tumor necrosis factor-α, thereby exacerbating the inflammatory response (Kanauchi et al., 2006; Lu et al., 2022). Meanwhile, *Megamonas* is considered a probiotic bacterium with potential contributions to maintaining an anti-inflammatory milieu through the regulation of the balance between regulatory T cells and T helper cell 17 (Shimizu et al., 2019; Yang et al., 2023).

*MiR-514a-3p* displayed associations with the abundance of three specific intestinal bacteria (*Lachnospiraceae_NK4A136_ group*, *Eubacterium_ruminantium_group*, and *Lachnospiraceae_ UCG.010*), in addition to four miRNAs (*miR-378c*, *miR-335-5p*, *miR-34c-5p*, and *miR-215-5p*). While direct correlations of *miR-514a-3p*, *miR-378c*, and *miR-335-5p* with FC have not been reported, their known functions and their potential relevance to FC pathogenesis offer valuable avenues for future investigation. Previous studies have indicated that *miR-514a-3p* and *miR-335-5p* may contribute to the proliferation, migration, and phenotypic switch of vascular smooth muscle cells (VSMCs) (Ma J. et al., 2022; Ma R. et al., 2022). Additionally, *miR-514a-3p* was identified as a novel tumor suppressor, with functional assays demonstrating its capacity to inhibit cell proliferation by targeting the epidermal growth factor receptor in clear cell renal cell carcinoma (Ke et al., 2017). Additionally, *miR-378c* has shown a promising value as a diagnostic and prognostic indicator for gastric cancer (Zhang et al., 2021). Notably, *miR-378c* can promote phenotypic modulation of VSMCs within atherosclerotic lesions by directly targeting the sterile alpha motif domain containing 1 (Tian et al., 2021).

Furthermore, a connection involving *miR-34c-5p*, *Eubacte rium_ruminantium_group*, *miR-215-5p*, *Lachnospiraceae_NK4A 136_group*, and *Lachnospiraceae_UCG.010* was also identified. Xu et al. (2021) demonstrated that M1 macrophage-derived exosomal *miR-34c-5p* could target the tyrosine kinase receptor/stem cell factor signaling pathway. This regulation influenced the expression profile of ICC in colonic tissues, ultimately restoring intestinal motility in an FC rat model. Additionally, Yao et al. (2020) investigated the role of *miR-215-5p* in the regulation of inflammation, suggesting its potential relevance to FC-related research. Furthermore, Cai et al. (2019) found that *miR-215-5p* might modulate cell autophagy by targeting the phosphoinositide 3-kinase/protein kinase B/mammalian target of rapamycin signaling pathway and the reactive oxygen species/mitogen-activated protein kinase signaling pathway. Given the established correlation between cell autophagy and the initiation and progression of FC (Wang et al., 2022), this association holds particular significance. In summary, these identified interactions within the present study provide valuable insights for further exploration and validation in animal models to elucidate their directional causality.

Although we recruited patients without severe anxiety, included FC patients still showed significantly higher SAS scores. The relationship between anxiety and FC is complex and multifaceted. Studies have shown that people with anxiety are more likely to experience gastrointestinal symptoms, including constipation; on the other hand, the embarrassment of living with FC can also exacerbate feelings of anxiety (Liu et al., 2021; Shiha et al., 2021). The gut microbiota has emerged as a key player in the bidirectional communication between the gut and the brain, known as the gut-brain axis (Margolis et al., 2021). Emerging evidence suggests that disturbances in the gut-brain axis may contribute to the development of both anxiety and FC (Jin et al., 2020; Westfall et al., 2021). Studies in animals have demonstrated that perturbations in the gut microbiota can lead to anxiety-like behaviors (Tao et al., 2024), while clinical research in humans has suggested that alterations in the gut microbiota may be associated with anxiety disorders (Wei et al., 2023). The gut microbiota could influence gut-brain axis through several mechanisms, including the production of serotonin and gamma-aminobutyric acid (Zhang W. et al., 2023), modulation of the immune response (Li et al., 2023), and regulation of the hypothalamic-pituitary-adrenal axis (Tsilimigras et al., 2018). In light of these findings, future studies could explore whether fecal miRNAs could be utilized as a potential target for modifying the gut microbiota to help manage anxiety and FC.

Currently, the etiology and pathogenesis of FC remain elusive and are primarily associated with aberrations in gastrointestinal motility, chronic intestinal inflammatory responses, diminished mechanosensitivity in the intestine, and abnormal brain-gut interactions (Wald, 2016; Vriesman et al., 2020). Intriguingly, functional predictions arising from this investigation have unveiled a significant enrichment of axon guidance pathways in miRNAs closely intertwined with the gut microbiota. Of note, glycans play pivotal roles in transmitting axonal signals, and enzymes responsible for glycan modifications finely regulate the information conveyed to axons, which can subsequently be interpreted by glycan-binding proteins (Sakamoto et al., 2021; Abad-Rodríguez et al., 2023). Enhanced glycan production may, in turn, augment the recruitment of certain bacteria (Kudelka et al., 2020). Consequently, these findings might shed light on a novel underlying mechanism by which miRNAs can selectively recruit particular microbes by orchestrating glycan biosynthesis and enhancing axon guidance function in the nervous system, ultimately retarding intestinal transit. Nevertheless, this mechanism warrants further comprehensive exploration.

Given the incomplete understanding of FC’s precise pathophysiology and the absence of specific biomarkers, FC is typically diagnosed based on clinical symptoms, often in conjunction with procedures such as enteroscopy (Aziz et al., 2020; Black et al., 2020). The diagnosis is confirmed after ruling out other organic gastrointestinal disorders, which can be challenging and may lead to misdiagnosis or undiagnosis (Sharma et al., 2021; Chiarioni et al., 2023; Sun et al., 2023). In this study, a combined diagnostic approach employing seven diagnostic biomarkers (*Oscillibacter*, *Escherichia.Shigella*, *UCG.002*, *miR-205-5p*, *miR-493-5p*, *miR-215-5p*, and *Lachnospiraceae_NK4A136_group*) yielded an impressive AUC value of 83.20%. This signifies a high predictive accuracy and holds significant promise for clinical application (Ren et al., 2019). This study introduces a novel diagnostic method for FC from the perspective of miRNA-gut microbiota interactions. Moreover, this non-invasive, cost-effective, and convenient fecal sample-based diagnosis provides an expedited and valuable clinical tool.

Nonetheless, several limitations warrant acknowledgment in this study. Firstly, the relatively small clinical sample size in this study may limit the detection of subtle miRNA expression differences between groups. Secondly, the study does not directly validate the causal relationships between these miRNAs and bacterial genera. Future study should consider isolating differentiated strains to verify its relevance with miRNAs. Thirdly, predictions derived from bioinformatics may not always precisely mirror real biological systems. Despite these constraints, the study’s elucidation of potential interactions between fecal miRNAs and the gut microbiota underscores their critical relevance for future inquiries into the pathogenesis of constipation.

## 4 Materials and methods

### 4.1 Ethics statement

The study was approved by the Ethics Committee of the Affiliated Hospital of Chengdu University of Traditional Chinese Medicine (Approval No. 2021KL-023). All subjects signed an informed consent form. All of them received a case report form and a fecal collection kit.

### 4.2 Study subject recruitment

A total of 30 patients with FC and 30 healthy volunteers were enrolled in the study. All patients with FC were recruited from the Outpatient Clinic of Anorectal and Digestive Departments of the Affiliated Hospital of Chengdu University of Traditional Chinese Medicine from February 2022 to December 2022. All patients with constipation were diagnosed by the specialist of the Anorectal or Digestive Department prior to enrollment. Inclusion criteria: patients aged 18–60 years regardless of gender; patients who met the Rome IV criteria (Palsson et al., 2020) for the diagnosis of FC and had a history of constipation for at least half a year; patients with a frequency of complete spontaneous bowel movements (CSBMs) ≤ 2 times per week, which lasted for more than 3 months (Yao et al., 2022); patients without special eating habits (vegetarian, vegan diet, or related to particular religious or social traditions) and mental disorders such as severe anxiety and depression [self-rating anxiety scale (SAS) or self-rating depression scale (SDS) standard score < 75 points]; the patients with exertion in defecation and dry stool at baseline [Bristol stool form scale (BSFS) ≤ 3 points]; patients who had no medication history for constipation, including intestinal microecologics, probiotics preparation, etc. Exclusion criteria: patients with a history of abdominal or anorectal surgery; patients with irritable bowel syndrome and organic or drug-induced constipation; patients with constipation secondary to endocrine, metabolic, or neurogenic constipation; patients with heart, liver, kidney damage or cognitive dysfunction, aphasia, or unable to cooperate with sampling and treatment; patients with other primary diseases caused by gut microbiota disorder (diabetes, obesity, migraine, etc.); pregnant or lactating patients, or patients with a pregnancy plan within the next 3 months. The health volunteers were mainly from society and online (WeChat Official Account and Moments). Inclusion criteria for healthy volunteers: aged 18–60 years regardless of gender; all subjects underwent a physical examination to ensure good physical health; no record of antibiotics, probiotics, yogurt, and other fermented dairy products during the baseline period; subjects not enrolled in other clinical studies, and signed the informed consent form. Exclusion criteria: following the special diet for other reasons (vegetarian, vegan diet, or related to particular religious or social traditions) were excluded; subjects with mental disorders such as severe anxiety and depression; subjects during pregnancy or lactation; subjects who cannot cooperate with blood and fecal sample collection.

The baseline clinical data of each subject, including gender, age, course of the disease, gastrointestinal symptoms, medication history, allergic history, and lifestyle habits (such as water intake and exercise level) was recorded by the investigator. The quality of life of all subjects was assessed using Patient-Assessment of Constipation Quality of Life (PAC-QOL), and the psychological and emotional disorders of patients were evaluated with SAS and SDS.

### 4.3 Fecal sample collection and storage

In the week before sampling, participants were instructed to maintain their regular diet (with no special changes compared with the previous); 72 h before sampling, participants were instructed to avoid intense physical activities such as running; and 24 h before sampling, it is mandatory to refrain from consuming coffee, chocolate, or any other caffeinated foods. Patients received the teaching video, as well as detailed written and oral instructions on how to collect feces using the Longsee fecal microbial genome protection solution kit (Guangdong Nanxin Medical Technology Co., Ltd.). The collection of two tubes of feces is required for the detection of gut microbiome and fecal miRNAs. Because it is difficult for FC patients to collect feces, the collection site could be chosen at home or in the hospital according to the specific situation. About 300 mg of formed stool from the middle section is collected, and then immediately transferred to a −80°C refrigerator in the hospital or temporarily frozen in the refrigerator at home. It should be quickly transferred to the researchers within 24 h for further sequencing analysis.

### 4.4 DNA isolation and 16S rRNA gene sequencing

The genomic DNA was extracted from the samples using CTAB- or SDS-based method, and then the purity and concentration of the DNA were detected by agarose gel electrophoresis. An appropriate amount of sample DNA was placed into a centrifuge tube and diluted to 1 ng/μL with sterile water. With the diluted genomic DNA as the template, PCR amplification was performed targeting the V3-V4 region of 16S rRNA using specific Barcode-tagged primers. The primer sequences of the 16S V34 region were as follows: forward (F): CCTAYGGGRBGCASCAG; R: GGACTACNNGGGTATCTAAT. The library was constructed using TruSeq^®^ DNA PCR-Free Sample Preparation Kit, and the constructed library was sequenced on the computer using the Illumina Hiseq platform (NovaSeq6000, China). All Effective Tags were clustered using the Uparse algorithm (Uparse v7.0.1001),^2^ and the obtained sequences were clustered into Amplicon Sequence Variants (ASVs) at 97% Identity (default). The Mothur method and SILVA138.1^3^ SSUrRNA database were employed for species annotation and analysis of ASVs sequences (threshold value set at 0.8∼1).

### 4.5 Analysis of species diversity, community structure, and differential microbes

The parameters Observed-species, Chao1, Shannon, Simpson, ACE, Goods-coverage, and PD_whole_tree were calculated using Qiime software (Version 1.9.1) to evaluate the α-diversity of the gut microbiota. The rank abundance curve, and species accumulation curve were plotted with R software (Version 4.2.1), and the difference in α-diversity between groups was analyzed with R software. The Unifrac distance was calculated using Qiime software (Version 1.9.1). Principal coordinate analysis (PCoA) and non-metric multi-dimensional scaling (NMDS) maps were plotted using R software to evaluate the β-diversity. The PCoA analyses were conducted with the ade4 package and ggplot2 package of R software, and NMDS analysis was done using the vegan package of R software. LEfSe analysis was performed with LEfSe software, and a linear discriminant analysis (LDA) score of 3.5 was set as the default screening value. The ANOSIM function of the vegan package in the R program was employed for ANOSIM analysis. The species showing significant differences between groups were subjected to a *t*-test and the graphs were drawn using R software.

### 4.6 MicroRNA extraction and sequencing

Fecal samples from six patients with FC and six healthy subjects were selected using stratified random sampling with the age as the stratifying variable to minimize the potential for any bias or confounding factors (Verhoef et al., 2014; Kho et al., 2016) (demographic characteristics of these subjects can be found in Supplementary Table 1). The total RNA was extracted from samples using the miRNeasy Mini Kit (Qiagen, Hilden, Germany). The purity and concentration of total RNA in the samples were determined by NanoDrop ND1000 (Agilent Technologies, Inc., CA). The miRNA sequencing was performed with the Illumina HiSeq 2000 sequencing system. The total RNA of all samples was used to construct the miRNA sequencing library, including the following steps: (1) 3’-linker connection; (2) 5′-linker connection; (3) cDNA synthesis; (4) PCR amplification; (5) Selection of a PCR amplification fragment with a size of about 135–155 bp (corresponding to a small RNA of 15 to 35 nt). The library was denatured into single-stranded DNA molecules, captured on Illumina flow cells (Illumina, San Diego, CA), and amplified into clusters *in situ*, followed by 51 cycles of final sequencing on Illumina NextSeq according to the manufacturer’s instructions. The expression profiles of miRNAs were statistically compared to the known genomes and obtained by the software miRdeep2. The mean value of counts per million reads (CPM) ≥ 1 was set as the threshold value of miRNA expression in each group, and the miRNAs that met the criteria were considered to be expressed in the subgroup and then subjected to statistical analysis.

### 4.7 Correlation analysis and co-occurrence network construction

Correlations among the frequency of completely autonomous defecation in a week, fecal trait scores, degree of defecation difficulty, PAC-QOL, SDS, SAS, gut bacterial genus, and differentially expressed microRNAs were analyzed using Spearman’s correlation coefficient. During the Spearman correlation analysis, the Spearman correlation coefficient values for species and environmental factors were first calculated with the corr.test function from the psych package in R software and then tested statistically for significance. The results were then visualized with the pheatmap function from the pheatmap package.

After obtaining the matrix of correlation coefficients between genus and miRNAs, the screening criteria were set as follows: (a) the weakly-correlated connections were removed with a cutoff value (>0.6) as the threshold; (b) node self-connections were filtered out; (c) connections with a node abundance less than 0.005% were removed; According to the filtered correlation coefficient values, a co-occurrence network was constructed using Cytoscape Version 3.9.1 software^4^ with bacterial genus or miRNAs as nodes and values as edges.

### 4.8 Random forest analysis

Random forest is a classical machine learning model based on the classification tree algorithm that classifies the data into training and testing sets, and then the classification function can be continuously trained with the training set to achieve the optimal classification performance, which is validated using the testing set. Random forest analysis was performed using the Random Forest package in R software, and the significant species were screened with Random-Forest scores. Afterward, the performance of each model was cross-validated (default 10-fold). Receiver operating characteristic (ROC) curves were drawn, and the area under the curve (AUC) was calculated to evaluate the diagnostic performance of the model.

### 4.9 Functional annotation of bacterial genus and microRNAs

For the gut bacterial genus, the functional gene compositions of the target bacteria were analyzed using PICRUSt software, Kyoto Encyclopedia of Genes and Genomes (KEGG) pathway data corresponding to ASVs were obtained, and functional differences were analyzed. For the differentially expressed microRNAs, we first performed target gene prediction with the multimiR package in R software. The target genes of the differentially expressed miRNAs were then mapped to individual nodes of the Gene Ontology (GO) database, and the number of genes in each node was counted. Subsequently, Fisher’s exact test was used to identify significantly enriched GO terms in the target genes of the differentially expressed miRNAs. Through this analysis, the molecular function (MF), cellular component (CC), and biological process (BP) of miRNA target genes were analyzed to reveal their biological functions. Finally, with KEGG Pathway as a unit, Fisher’s exact test was applied to identify the pathways significantly enriched by the target genes of the differentially expressed miRNAs, to identify the dominating signal transduction pathways and biochemical metabolic pathways in which they were involved.

### 4.10 Data analysis

SPSS 23.0 software was adopted for statistical analysis of the difference between the two groups of clinical parameters. The gender ratio was examined using 2 × 2 chi-square text. The normality of all data was tested by the Shapiro–Wilk test, with *p* > 0.05 indicating normal distribution, and the homogeneity of variance was examined using one-way analysis of variance (ANOVA) with *p* > 0.05 deemed as an equal variance. The continuous variables obeying normal distribution and equal variance were represented by $\bar{x}$ ± *S*, and compared between the two groups by the independent sample *t*-test; otherwise, the continuous variables were represented as median (M), interquartile range (IQR), and *Wilcoxon Mann-Whitney U* test was employed for their comparison between the two groups. All sequenced data were analyzed with R software. All statistical results were considered statistically significant with *p* < 0.05.

## 5 Conclusion

In summary, this comprehensive study highlights notable disruptions in the gut microbiota composition in individuals affected by FC. Furthermore, our investigation unveiled dozens of fecal miRNAs that are differentially regulated in FC patients. Notably, we identified interactions between the abundance of seven specific bacterial genera and the expression of nine fecal miRNAs, strongly implicating fecal miRNAs as potential mediators of the host-microbiome interplay in FC. This work provides a first systems-level map of the association between microbes and fecal miRNAs in FC, and subsequent investigations should consider isolating differentiated strains to verify their causal relevance with miRNAs.

## Data availability statement

The datasets presented in this study can be found in online repositories. The names of the repository/repositories and accession number(s) can be found below: NCBI, PRJNA1048321.

## Ethics statement

The study was approved by the Ethics Committee of the Affiliated Hospital of Chengdu University of Traditional Chinese Medicine (Approval No. 2021KL-023). All subjects signed an informed consent form. The studies were conducted in accordance with the local legislation and institutional requirements. The participants provided their written informed consent to participate in this study.

## Author contributions

JY: Formal analysis, Writing—original draft. XY: Formal analysis, Writing—original draft. YQL: Formal analysis, Writing—review and editing. YC: Project administration, Writing—review and editing. XX: Methodology, Writing – review and editing. SZ: Validation, Writing—review and editing. WZ: Validation, Writing—review and editing. LW: Project administration, Writing—review and editing. MC: Project administration, Writing—review and editing. FZ: Conceptualization, Writing—review and editing. YL: Conceptualization, Writing—review and editing.

## Footnotes

1
http://www.mirbase.org/

2
http://www.drive5.com/uparse/

3
http://www.arb-silva.de/

4
http://www.cytoscape.org
