# Supplementary material for: Altered gut microbial profile is associated with differentially expressed fecal microRNAs in patients with functional constipation
Source: Front Microbiol. 2024 Jan 11;14:1323877. doi: 10.3389/fmicb.2023.1323877 (PMC10808787; doi:10.3389/fmicb.2023.1323877)
Supplement: Supplementary file 1 [file Data_Sheet_1.zip › Table 1.DOCX]

**Anomalous Gut Microbiota Composition Correlates with Disparate Expression Patterns of Fecal MicroRNAs in Individuals Diagnosed with Functional Constipation**


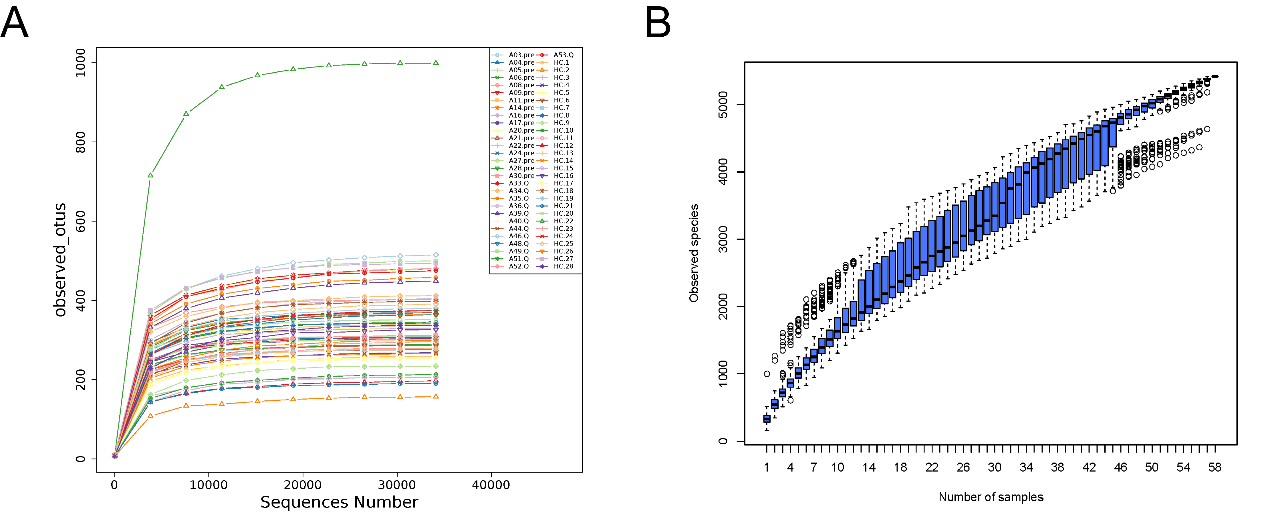


**Figure S1**. The rarefaction curves and species accumulation boxplots in FC and HC. (**a**) The rarefaction curves and (**b**) Species accumulation curves.


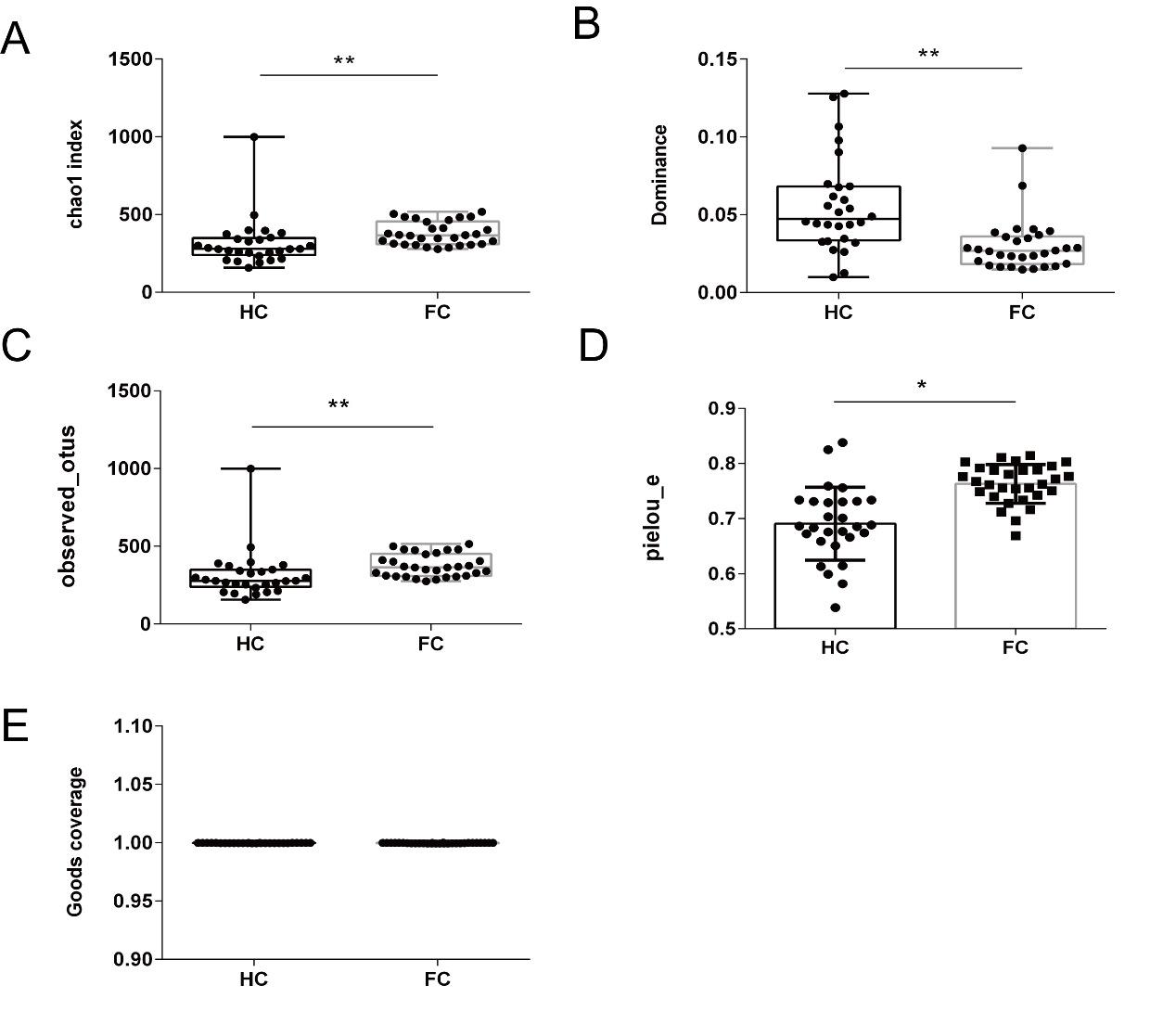


**Figure S2**. Differential analysis of the remaining α-diversity indices between the FC and HC groups. (**a**) Chao1 index, (**b**) Dominance, (**c**) Observed_otus, (**d**) pielou_e, and (**e**) Goods coverage.


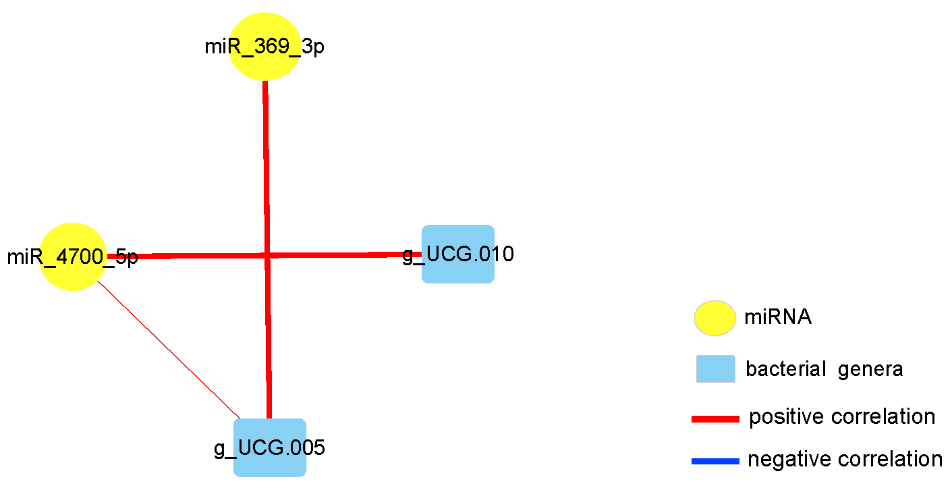


**Figure S3**. The interaction cluster among miR-4700-5p, miR-369-3p, UCG.005, and UCG.010.


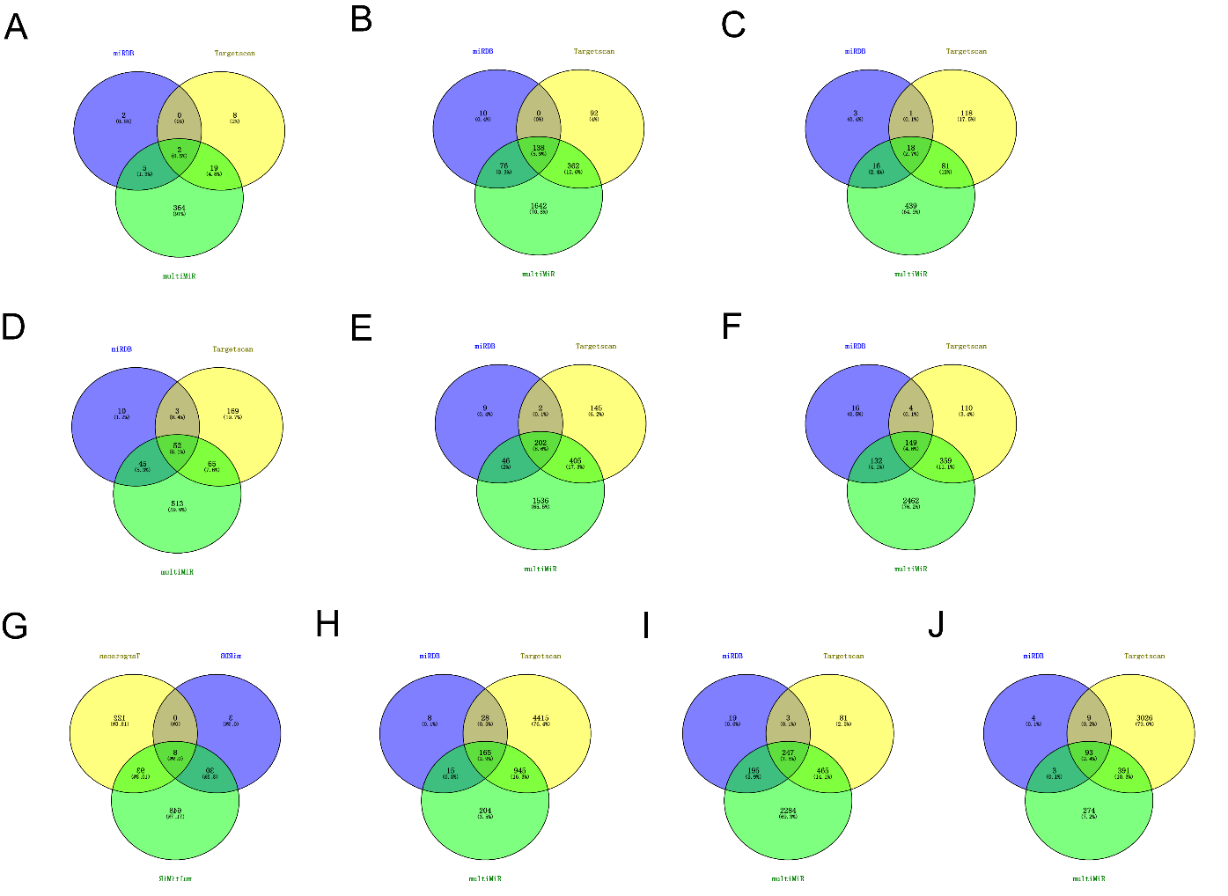


**Figure S4**. Venn analysis of DE miRNAs in the prediction of target genes.
