# Supplementary material for: Altered gut microbial profile is associated with differentially expressed fecal microRNAs in patients with functional constipation
Source: Front Microbiol. 2024 Jan 11;14:1323877. doi: 10.3389/fmicb.2023.1323877 (PMC10808787; doi:10.3389/fmicb.2023.1323877)
Supplement: Supplementary file 1 [file Data_Sheet_1.zip › Table 2.DOCX]

**Altered Gut Microbial Profile is Associated with Differentially Expressed Fecal MicroRNAs in Patients with Functional Constipation**

| **Table S1. Demographics and characteristics of subjects for miRNA analysis** | | | |
| --- | --- | --- | --- |
|  | Healthy subjects | FC patients | *p*-value |
| Subjects (n) | 6 | 6 | N/A |
| Age (years) | 31.33±9.50 | 32.66±10.11 | 0.819 |
| Sex, male/female | 0/6 | 0/6 | N/A |
| Body mass index (kg/m^2^) | 21.25±1.98 | 21.46±2.59 | 0.878 |
| Disease duration (months) | / | 79.00±47.76 | N/A |
| Average of CSBMs per week (n) | 6.83±1.33 | 1.50±1.05 | 0.000016 |
| Average of SBMs per week (n) | 7.50[1.25] | 2.50[3.00] | 0.004 |
| Straining during defecation | 0.00[0.08] | 1.00[1.12] | 0.015 |
| BSFS scores | 4.17[0.64] | 2.92[2.33] | 0.041 |
| PAC-QOL scale | 1.17[0.17] | 2.45[0.89] | 0.002 |
| Self-rating depression scale | 38.13±5.58 | 42.29±11.11 | 0.431 |
| Self-rating anxiety scale | 36.88±9.93 | 42.92±9.96 | 0.317 |

FC, functional constipation; CSBMs, complete spontaneous bowel movements; SBMs, spontaneous bowel movements; BSFS, bristol stool form scale; PAC-QOL, patient-assessment of constipation quality of life.

| **Table S2 The correlation between bacterial genera and constipated-indicators** | | | |
| --- | --- | --- | --- |
| Taxonomy | *r*-value | *p*-value | *FDR* corrected *p*-value |
| **CSBMs** | | | |
| UCG.010 | -0.415 | 0.009 | 0.023 |
| Lachnospiraceae_UCG.010 | -0.480 | 0.000 | 0.007 |
| Escherichia.Shigella | -0.372 | 0.014 | 0.043 |
| **SBMs** | | | |
| Lachnospiraceae_UCG.010 | -0.462 | 0.011 | 0.010 |
| Escherichia.Shigella | -0.389 | 0.013 | 0.034 |
| UCG.010 | -0.402 | 0.004 | 0.028 |
| UCG.005 | -0.367 | 0.033 | 0.046 |
| UCG.002 | -0.428 | 0.000 | 0.018 |
| **BSFS scores** | | | |
| Parabacteroides | 0.334 | 0.018 | 0.041 |
| **Straining during defecation** | | | |
| Oscillibacter | 0.352 | 0.017 | 0.037 |
| Bacteroides | 0.321 | 0.005 | 0.024 |
| Flavonifractor | 0.323 | 0.003 | 0.032 |
| g_Escherichia.Shigella | -0.340 | 0.000 | 0.016 |
| Eubacterium_ruminantium_group | -0.448 | 0.000 | 0.013 |
| Barnesiella | -0.341 | 0.023 | 0.045 |
| UCG.010 | -0.426 | 0.000 | 0.019 |
| Megamonas | -0.375 | 0.000 | 0.011 |
| **PAC-QOL scale** | | | |
| Megamonas | -0.407 | 0.009 | 0.026 |
| Lachnospiraceae_NK4A136_group | 0.311 | 0.026 | 0.045 |
| Colidextribacter | 0.356 | 0.014 | 0.033 |
| **SDS** | | | |
| Lachnospiraceae_UCG.010 | -0.450 | 0.000 | 0.013 |

CSBMs, complete spontaneous bowel movements; SBMs, spontaneous bowel movements; BSFS, Bristol stool form scale; PAC-QOL, Patient-Assessment of Constipation Quality of Life; SDS, self-rating depression scale.

| **Table S3 The correlation between DE miRNAs and FC-associated bacterial genera** | | | |
| --- | --- | --- | --- |
| Taxonomy | *r*-value | *p*-value | *FDR* corrected *p*-value |
| ***hsa-miR-184*** | | | |
| Oscillibacter | 0.771 | 0.017 | 0.042 |
| Escherichia.Shigella | -0.829 | 0.008 | 0.033 |
| Megamonas | -0.771 | 0.015 | 0.042 |
| ***hsa-miR-205-5p*** | | | |
| Escherichia.Shigella | -0.928 | 0.000 | 0.008 |
| Megamonas | -0.928 | 0.000 | 0.008 |
| Lachnospiraceae_NK4A136_group | -0.928 | 0.000 | 0.008 |
| Eubacterium_ruminantium_group | -0.794 | 0.021 | 0.049 |
| Lachnospiraceae_UCG.010 | -0.899 | 0.001 | 0.015 |
| Oscillibacter | 0.812 | 0.026 | 0.050 |
| ***hsa-miR-493-5p*** | | | |
| Eubacterium_ruminantium_group | -0.765 | 0.021 | 0.047 |
| ***hsa-miR-514a-3p*** | | | |
| Lachnospiraceae_NK4A136_group | -0.778 | 0.022 | 0.049 |
| Eubacterium_ruminantium_group | -0.823 | 0.013 | 0.044 |
| Lachnospiraceae_UCG.010 | -0.778 | 0.015 | 0.049 |
| ***hsa-miR-378c*** | | | |
| UCG.002 | 0.771 | 0.008 | 0.033 |
| Lachnospiraceae_NK4A136_group | -0.829 | 0.020 | 0.042 |
| ***hsa-miR-141-3p*** | | | |
| UCG.002 | 0.880 | 0.003 | 0.021 |
| ***hsa-miR-4700-5p*** | | | |
| UCG.005 | 0.943 | 0.001 | 0.005 |
| UCG.010 | 0.771 | 0.011 | 0.033 |
| ***hsa-miR-369-3p*** | | | |
| UCG.005 | 0.771 | 0.011 | 0.033 |
| ***hsa-miR-335-5p*** | | | |
| Eubacterium_ruminantium_group | 0.812 | 0.001 | 0.050 |
| ***hsa-miR-34c-5p*** | | | |
| Lachnospiraceae_NK4A136_group | 0.771 | 0.011 | 0.033 |
| Lachnospiraceae_UCG.010 | 0.771 | 0.011 | 0.033 |
| ***hsa-miR-215-5p*** | | | |
| Lachnospiraceae_NK4A136_group | 0.771 | 0.011 | 0.033 |
| UCG.002 | -0.886 | 0.001 | 0.019 |

| **Table S4 The detailed information of nodes in the network** | | | | | |
| --- | --- | --- | --- | --- | --- |
| **Nodes_id** | **degree** | **weight_degree** | **closeness_centrality** | **betweenness_centrality** | **eigenvector_centrality** |
| hsa-miR-184 | 3 | 0.146135110370679 | 0.8070062933714 | 0 | 0.300395896429983 |
| hsa-miR-205-5p | 8 | 0.245860050358174 | 1.54853778866672 | 50 | 0.683953338192448 |
| hsa-miR-493-5p | 3 | 0.0837093106190542 | 1.08478755910612 | 6 | 0.214309618839751 |
| hsa-miR-514a-3p | 9 | 0.357028934361754 | 1.35630828254349 | 16 | 1 |
| hsa-miR-378c | 5 | 0.250998991844022 | 1.0912446317405 | 0 | 0.741954613269805 |
| hsa-miR-141-3p | 2 | 0.0789601173952564 | 0.772314433485278 | 0 | 0.19008929102332 |
| hsa-miR-4700-5p | 2 | 0.0603788421491045 | 8.28104650906122 | 2 | 0 |
| hsa-miR-369-3p | 1 | 0.05584872969596 | 4.30190523696492 | 0 | 0 |
| hsa-miR-335-5p | 3 | 0.0696007395255223 | 1.08550500776266 | 8 | 0.198951580653392 |
| hsa-miR-34c-5p | 4 | 0.157216895294947 | 1.12514620967383 | 0 | 0.601732483075986 |
| hsa-miR-215-5p | 6 | 0.234733469143783 | 1.16644327792117 | 25 | 0.644747230643238 |
| UCG.002 | 3 | 0.0906756968158914 | 0.960692460449231 | 14 | 0.242526916182455 |
| UCG.005 | 2 | 0.0603788421491045 | 8.28104650906122 | 2 | 0 |
| UCG.010 | 1 | 0.05584872969596 | 4.30190523696492 | 0 | 0 |
| Escherichia.Shigella | 4 | 0.125128940837613 | 1.32096802322701 | 14 | 0.269006725486973 |
| Megamonas | 6 | 0.195879108405735 | 1.4231649340008 | 38 | 0.565396037383224 |
| Lachnospiraceae_NK4A136_group | 7 | 0.296970663738894 | 1.48916740090757 | 27 | 0.931346018925104 |
| Eubacterium_ruminantium_group | 5 | 0.189455036945501 | 1.39792823473587 | 18 | 0.414035426514651 |
| Lachnospiraceae_UCG.010 | 7 | 0.275912600049535 | 1.42628506557684 | 20 | 0.831891408816645 |
| Oscillibacter | 5 | 0.224706278123703 | 1.06798446107191 | 0 | 0.502850944521578 |

| **Table S5 The detailed information of edges in the network** | | | | | | |
| --- | --- | --- | --- | --- | --- | --- |
| **Edges** | | |  | **weight** | **correlation** | **betweenness_centrality** |
| **Sources** |  | **Targets** |  |  |  |  |
| hsa-miR-184 |  | Escherichia.Shigella |  | 0.034438 | -0.03444 | 15 |
| hsa-miR-184 |  | Megamonas |  | 0.055849 | -0.05585 | 0 |
| hsa-miR-184 |  | Oscillibacter |  | 0.055849 | 0.055849 | 0 |
| hsa-miR-205-5p |  | hsa-miR-514a-3p |  | 0.049026 | 0.049026 | 5 |
| hsa-miR-205-5p |  | hsa-miR-378c |  | 0.051426 | 0.051426 | 0 |
| hsa-miR-205-5p |  | Escherichia.Shigella |  | 0.030312 | -0.03031 | 0 |
| hsa-miR-205-5p |  | Megamonas |  | 0.007111 | -0.00711 | 48 |
| hsa-miR-205-5p |  | Lachnospiraceae_NK4A136_group |  | 0.007111 | -0.00711 | 36 |
| hsa-miR-205-5p |  | Eubacterium_ruminantium_group |  | 0.047026 | -0.04703 | 0 |
| hsa-miR-205-5p |  | Lachnospiraceae_UCG.010 |  | 0.01338 | -0.01338 | 26 |
| hsa-miR-205-5p |  | Oscillibacter |  | 0.040468 | 0.040468 | 0 |
| hsa-miR-493-5p |  | hsa-miR-514a-3p |  | 0.02488 | 0.02488 | 12 |
| hsa-miR-493-5p |  | hsa-miR-335-5p |  | 0.000305 | -0.0003 | 15 |
| hsa-miR-493-5p |  | Eubacterium_ruminantium_group |  | 0.058524 | -0.05852 | 0 |
| hsa-miR-514a-3p |  | hsa-miR-378c |  | 0.053438 | 0.053438 | 3 |
| hsa-miR-514a-3p |  | hsa-miR-335-5p |  | 0.028828 | -0.02883 | 0 |
| hsa-miR-514a-3p |  | hsa-miR-34c-5p |  | 0.028828 | -0.02883 | 3 |
| hsa-miR-514a-3p |  | hsa-miR-215-5p |  | 0.028828 | -0.02883 | 15 |
| hsa-miR-514a-3p |  | Lachnospiraceae_NK4A136_group |  | 0.053438 | -0.05344 | 1 |
| hsa-miR-514a-3p |  | Eubacterium_ruminantium_group |  | 0.036325 | -0.03633 | 8 |
| hsa-miR-514a-3p |  | Lachnospiraceae_UCG.010 |  | 0.053438 | -0.05344 | 0 |
| hsa-miR-378c |  | hsa-miR-215-5p |  | 0.055849 | -0.05585 | 2 |
| hsa-miR-378c |  | UCG.002 |  | 0.055849 | 0.055849 | 2 |
| hsa-miR-378c |  | Lachnospiraceae_NK4A136_group |  | 0.034438 | -0.03444 | 8 |
| hsa-miR-141-3p |  | hsa-miR-215-5p |  | 0.060825 | -0.06082 | 0 |
| hsa-miR-141-3p |  | UCG.002 |  | 0.018135 | 0.018135 | 15 |
| hsa-miR-4700-5p |  | UCG.005 |  | 0.00453 | 0.00453 | 4 |
| hsa-miR-4700-5p |  | UCG.010 |  | 0.055849 | 0.055849 | 3 |
| hsa-miR-369-3p |  | UCG.005 |  | 0.055849 | 0.055849 | 3 |
| hsa-miR-335-5p |  | Eubacterium_ruminantium_group |  | 0.040468 | 0.040468 | 16 |
| hsa-miR-34c-5p |  | hsa-miR-215-5p |  | 0.016692 | 0.016692 | 4 |
| hsa-miR-34c-5p |  | Lachnospiraceae_NK4A136_group |  | 0.055849 | 0.055849 | 6 |
| hsa-miR-34c-5p |  | Lachnospiraceae_UCG.010 |  | 0.055849 | 0.055849 | 2 |
| hsa-miR-215-5p |  | UCG.002 |  | 0.016692 | -0.01669 | 26 |
| hsa-miR-215-5p |  | Lachnospiraceae_NK4A136_group |  | 0.055849 | 0.055849 | 18 |
| Escherichia.Shigella |  | Megamonas |  | 0.00453 | 0.00453 | 28 |
| Escherichia.Shigella |  | Oscillibacter |  | 0.055849 | -0.05585 | 0 |
| Megamonas |  | Lachnospiraceae_NK4A136_group |  | 0.055849 | 0.055849 | 0 |
| Megamonas |  | Lachnospiraceae_UCG.010 |  | 0.055849 | 0.055849 | 0 |
| Megamonas |  | Oscillibacter |  | 0.016692 | -0.01669 | 15 |
| Lachnospiraceae_NK4A136_group |  | Lachnospiraceae_UCG.010 |  | 0.034438 | 0.034438 | 0 |
| Eubacterium_ruminantium_group |  | Lachnospiraceae_UCG.010 |  | 0.007111 | 0.007111 | 27 |
| Lachnospiraceae_UCG.010 |  | Oscillibacter |  | 0.055849 | -0.05585 | 0 |

| **Table S6 Target genes in the Venn diagram intersections of miRNAs associated with microbiomes** | |
| --- | --- |
| **miRNA ID** | **Predicted target genes** |
| *hsa-miR-184* | NUS1, EPB41L5 |
| *hsa-miR-205-5p* | ESRRG, KPNA1, BTBD3, MMD, PTPRM, ZEB1, MGRN1, CDC27, FZD3, SIPA1L1, DSC1, VEGFA, RORA, QKI, ANKRD50, PAX9, RTN3, CADM1, TNFAIP8, ETNK1, LYSMD3, MTF1, ADAMTS9, ZBTB20, CMTM4, ZFYVE16, SORBS1, CDK19, EVA1C, EPB41, SLC35A1, RBPMS2, LRP1, TP53BP2, AMOT, BICC1, TNPO1, CSF1, CALCRL, CALU, PTK7, DLG2, RAP2B, MAGI1, GXYLT1, SLC35B3, NKD1, SH3GL3, PLCB1, CFL2, EZR, VTI1B, PSD3, AXIN2, TBX3, SUSD1, NEU1, HS3ST1, CDH11, ACSL1, ERRFI1, LPCAT1, MGA, CDK14, PHYHIPL, SLC19A2, RNF4, ROCK2, MSI2, ZNF652, RAB11FIP1, LIN9, LHFPL2, RBM47, SATB2, PROX1, MAGI2, STRBP, SMAD4, CLTC, PPP1R15B, ERBB3, HSPA13, SERTAD2, CHN1, PRKCE, TAPT1, CCNJ, SGMS1, SPRY1, C12orf29, LAMC1, NOTCH2, PJA2, CLDN11, AP1G1, RUNX2, LCOR, TM9SF2, INPPL1, C11orf86, NACC2, B4GALT5, NECAP1, FRK, YAP1, PDE3B, RPS6KA3, DSC2, AAK1, MICAL2, HERC3, SBF2, SMAD1, NFIB, FOXF1, ERBB4, MARCKS, NSF, PTPRJ, DUSP7, HSD17B11, ENC1, NAA25, FBXO22, TRAK2, ZHX3, ENPP4, LRRK2, TAOK1, AP1AR, CPEB2, P2RY1, SECISBP2L, ZNF800, NFAT5, SLC4A4, LRP6 |
| *hsa-miR-215-5p* | MTMR4, FRMD4B, WDR44, RAB2A, NKAIN2, ZBTB34, SRSF6, ZEB2, DYRK3, NIPAL1, BHLHE22, OSBPL10, EREG, RPAP2, PDP1, LPAR4, WNK1, ARFGEF1 |
| *hsa-miR-335-5p* | SMARCA2, CALU, TMEM59, HAND1, PRKAA2, MTMR4, CERS5, FBXO28, KDM2B, FAM107B, PSD3, KAT7, CASP7, F13A1, HOXD8, SORCS1, NRXN1, POU5F1, CCNF, VAPA, GJA5, SEPHS1, FMN1, FAM131B, NCKAP5, KDM4C, CNOT7, PLEKHA8, CHFR, ARHGAP18, PGM3, EIF5A2, PTPRB, RPRM, RASA1, SREK1IP1, DAAM1, UBE2G1, POU2F3, ZRANB1, HPCAL4, ZMPSTE24, CAMKK2, ARGLU1, NAA25, CEP350, RBFOX2, SNIP1, NXPH2, GLYR1, APTX, SECISBP2L |
| *hsa-miR-34c-5p* | ASIC2, MDM4, MPP2, ADAM22, BCL2L13, FKBP1B, CACNA1E, ZNF281, KLF4, NRN1, FOXJ2, TANC2, RCAN1, FAM117B, RALGPS1, SHKBP1, NUMBL, AMER1, CPLX2, PURB, FAM76A, SERPINF2, DNAJC16, SAR1A, HNF4A, RAP1GDS1, AXL, GREM2, HTR2C, JAG1, INA, FUT8, HSPA1B, ABR, LGR4, MCIDAS, E2F5, TMEM164, PPARGC1B, TMEM109, ELMOD1, TBL1XR1, FUT9, SLC25A27, LEF1, PEA15, TGIF2, PLAG1, CDK6, PPP2R3A, CNOT6, MYCN, DLL1, WASF1, RRAS, RGS17, POGZ, ACSL4, METAP1, STX17, ACBD3, TAF4B, SDK2, PDXK, PKP4, MET, PPP1R10, NCEH1, KCNK3, FRMD5, CELF3, NOTCH1, PITPNC1, GPR158, AHCYL2, SLC4A7, PLEKHH2, TNRC18, SYNJ1, SGPP1, TSN, LMAN1, KIAA1217, ASB1, ZNF304, TMEM255A, NAV1, PPP1R16B, CUEDC1, PGM1, ARID4B, VCL, ZDHHC17, SATB2, FAM167A, TAF5, GMNC, ZNF644, ERGIC1, PLOD1, ARHGAP1, MYH9, BRPF3, FOXN2, SLC44A2, CERS6, ZMYM4, ZBTB20, NAV3, PPP1R11, GABRA3, TMEM200B, SEMA4B, MLLT3, CLCN3, STRN3, DPYSL4, NOTCH2, ANKS1A, IL6R, MGAT5B, HCN3, LDHA, UBP1, CACNB3, RELN, FBXO30, BMP3, SHOC2, AGO4, NPNT, NRIP3, ZFHX4, VAMP2, TMEM184B, FLOT2, PACS1, RPS6KA4, LMAN2L, RTN4RL1, TRIM67, XPO5, ACSL1, GPR22, DGKZ, DAGLA, ZDHHC16, UNC13C, STK38L, DIXDC1, GMFB, CTNND2, UHRF2, NOS1AP, ANKRD52, PNOC, E2F3, PDE7B, SNTB2, CCNE2, MLLT1, ANK2, FGD6, GOLPH3L, ADO, TOB2, LHX2, ADIPOR2, CA7, SYT1, XYLT1, RPS6KL1, ATMIN, RFX3, EML5, DCAF7, TRANK1, BNC2, PDGFRA, DAAM1, GALNT7, RRAGC, C14orf28, SMIM15, SCN2B, SNX15, OSGIN2, BRINP1, GLCE, MAP2K1, TPD52, CBFA2T3, CLOCK, PPFIA1, ADD2, SHISA7, ARHGAP26, KITLG, ANK3, FOXP1, SIDT2, MGAT4A |
| *hsa-miR-369-3p* | NR2F2, CEBPA, LTBP1, ZEB1, DMD, DLG3, ELOVL7, ZNF423, ZNF281, ADCY5, HOMER1, RBPJ, RFX4, TCF4, LRCH2, UBA6, PHACTR3, FBXL3, TENM1, EYA1, PAQR3, HCN1, GOPC, KLF7, PAPPA, CAPZA1, PPM1A, CFL2, SEZ6, PTPN12, STXBP5, BACE1, ARHGAP5, PDE4D, RORA, PARP8, RNF38, YTHDF3, NCOA2, TLE4, GLG1, BIRC3, CDYL2, RASSF8, FNIP1, FAF2, RC3H1, FAM135A, TBC1D9, RNF214, HOXB3, ZNF771, SP3, FOXN2, PHACTR2, CHSY1, MON2, RAB5B, TBX4, HSPH1, MBNL2, YAF2, ADNP, MAP2, ZFHX4, DENND4A, GRAMD1B, SKI, BMP2, SCN5A, ZNF521, FZD5, PDE8B, PRRC2C, CCNE2, MYLK4, CAPZA2, BICD2, ADAM10, PCDHA8, PELI1, CEP350, CEBPB, LIN7A, NCK1, INHBB, FLRT3, FEZF1, DESI1, JMJD1C, WBP4, MGAT4A, KIF20A, HAO1, RANBP9, CHD7, NUMBL, DYRK1A, NIPBL, RAB10, WDR20, SEMA5A, NF1, EP300, PNRC1, C11orf58, RGS7BP, RAI1, EFNA5, ZSWIM6, HES1, SEC23A, OCIAD1, GJC1, PTPN21, SETD2, MED13, PLXNA2, LCOR, SMAD6, USP30, FOXO1, UBAP1, CDK19, STK38L, NPY1R, ATF2, PCDH10, LCA5, SPAG7, CBX4, ARL2BP, JMY, ADAMTS3, RNF44, PAXIP1, ACLY, DR1, MCFD2, ARID2, TLL2, TMEM108, TNRC6A, DSG3, TRAPPC8, RYBP, GBX2, VEGFC, TRPV3 |
| *hsa-miR-378c* | TSPAN17, RAB10, METTL4, KIAA1522, ZFPM2, OTUB2, NPAS4, CREBRF |
| *hsa-miR-4700-5p* | PPP1R1B, PSMD5, CCDC178, NHLH1, HCCS, RANBP1, DCTN5, PCDHGA5, PCDHGA11, HEYL, POU3F3, PCDHGC3, CIITA, CASKIN1, ARFIP1, SUPT7L, FAM104B, MKRN2, ITPRIP, PXN, NR6A1, SERPINA1, EFCC1, TET3, SMYD1, IQSEC3, PADI2, LYPD6, RNF170, ARL10, STX17, VTI1A, VSTM4, PRKAR1B, APOM, KCNK3, NAA40, SSX1, SALL1, LASP1, EGLN3, ACACA, RGSL1, MEIS2, PRX, SRCAP, ABCC10, CELF6, CIC, DGAT2, PCDHGA3, ZNF687, GGA3, TRIM71, TSPAN11, SUSD2, CLSTN1, SAP30BP, SEMA3F, PCDHGB4, SLC25A14, NUMA1, PCDHGA9, DMWD, ZNF704, PRKCG, LOXHD1, UNC5B, SPIB, RARG, TBC1D13, ATXN1L, BCL2L1, BBIP1, PCDHGB3, SNX12, PCDHGB6, DAGLA, NAV1, PPP1CA, TJP1, NAGA, THRA, RASSF4, SLC6A9, SLC38A7, SGCD, MS4A15, CCSER2, GLIPR1, TTF2, TSPAN2, ZNF106, CAPN12, TMEM127, PCDHGC4, TEAD1, IL6R, NRIP2, LZTS1, GALNT15, KCNJ10, POPDC3, TBKBP1, MAT1A, NCDN, XPO7, FAM228B, RARB, CPLX2, MAT2A, MAPK1, PCDHGB1, FBXO41, ELMOD1, WDR72, SLC27A4, DNALI1, PCDHGA10, CDC42EP1, PCDHGA4, KDELR1, ATF6B, PPP1R10, FBLN1, CALN1, MLLT6, PCDHGA2, OSBPL7, CNGB1, SAMD12, SOX6, CNTN2, STAM2, FUT11, GLP1R, SLC34A2, PCDHGA12, ITGA5, PCDHGA6, RNASE13, KSR2, PCDHGB2, CLDN19, PCDHGB7, ARID3B, ANKIB1, PCDHGA8, C17orf107, BAK1, SLC17A2, PCDHGA1, GAB2, ANKRD20A1, AAK1, ENTPD3, PCDHGC5, FOXN3, SORT1, C2CD2L, S1PR2, PCDHGA7, EHD3, ZNF831, GIGYF1 |
| *hsa-miR-493-5p* | NUDT4, ZNF148, GGACT, ZNF384, MEF2C, NCOA7, RALGPS1, AKAP1, PURB, KDM2B, TCF4, GABRA5, ZMYND8, BBX, DCUN1D1, VASH2, LMO3, ZFAND5, FBXL3, PSD3, GPR176, GDNF, GCLC, NCOA1, NAB1, PRKD3, BCL7A, KDM6A, NEO1, HSPA12A, ARHGAP12, GOLM1, DAB2IP, ITPRIPL2, ARHGAP44, TCF7L2, NR2C2, GATA2, AHDC1, ATAD2, ADAM23, CADM2, CYTH3, DHX36, MLLT10, CREB1, SP1, REEP1, LIMCH1, GTF2IRD1, DLL1, ANKRD17, GDF6, HIF1A, DGKI, CDH11, ARHGAP5, ARID1B, PARP8, SERPINB7, GRM5, PPP2R2C, GRID2, SALL1, MYT1L, LARP4, TNRC6B, NDNF, ATP2A2, RGS10, PIK3C3, CELF2, NAV2, FNIP1, NDUFC2, MBD5, DACH1, CITED2, CTDSPL2, RAPGEF2, PRDM12, SVEP1, TSC22D2, ANKRD61, SERTAD2, SP3, AFF3, AP3S1, CLSTN1, FBXO33, TGFBRAP1, CXADR, ITGB1, LRP12, SCAMP2, MBNL2, FUBP1, SBNO1, GABRG2, DLG2, THSD7A, SH2B3, KIAA2026, UBE2G1, VAMP2, YIPF4, RXRA, HMGCR, CAMK2N1, PUM2, PDS5A, BTG1, TMEM106B, SEC63, FOXJ3, OSBPL6, SNRPB2, WNT5A, VPS13D, CPNE3, DIP2C, SCN1A, E2F3, TRIP10, BCOR, GNPTAB, TRHDE, MYCBP2, PIK3R1, CARM1, INSM2, GAS1, ANKRD12, TTC7B, DYRK4, TIA1, USP7, CKS2, MBNL3, RPS6KA5, AK5, PAPSS2, PANK3, RASL11B, KIAA1671, ANO4, L1CAM, NCL, CTNND2, ZMIZ1, PTCH1, UBE2V2, FBRSL1, DENND4C, ARFGEF1, RASAL2, NFKBIA, SAT1, HIPK1, AXIN1, NDST1, BNC2, PPP2R5D, EFNA3, TBC1D22B, SLC25A12, RAB10, BASP1, RAB5C, STARD13, PDPK1, TNFAIP8, STAM, ZNF385B, ANK2, PHF12, MAP3K2, UNC5C, TOX3, NCAPH, MEMO1, DIP2A, NPAS3, KCNK1, LRIG1, CDH2, PREX1, SRPX, MTSS1, NECAB1, MAP3K1, FMNL2, SLC22A23, SLC10A7, KIF18A, GJC1, SNX9, TMEM170B, UCK2, HMGCS1, ZMYM5, MED13L, ATAD2B, IRX3, FBXW11, LRRC8C, EOMES, LTBP3, MGAT3, HP1BP3, ACAP2, MED13, VSX2, SHOC2, NRXN3, EPHA7, KCNJ6, KIAA0408, GSKIP, PGRMC2, BMPR1A, ATAD5, WASF3, CWC25, HS3ST3B1, HIVEP2, ABTB2, HK2, RBBP5, SIN3A, FZD4, PPP1CC, SRGAP1, ACTR1B, USP32, SCRN1, ZIC2, PAIP1, ZNF638, CYFIP2, KPNA4, SMG1, TNFSF11, COG3, IL1A, ENDOD1, PAG1 |
| *hsa-miR-514a-3p* | BTBD1, AFF4, TCF12, CABLES1, HIPK3, TAC1, NCOA7, CHD7, JAM2, PCNP, TSPAN9, C7, PCCA, ECE1, ARHGEF9, USP27X, PPP1R12A, PEG3, EML6, HECTD2, SUCLG2, ANO5, ERC1, STARD7, NPAS3, DLK1, DCLK3, RABEP1, MS4A3, RBM27, CAPSL, SVIL, REPS2, CD200R1, DEFB132, ZNF282, PSMA2, VGLL3, FHAD1, FAM8A1, RBX1, PTEN, GULP1, ZFHX3, HELZ, BBIP1, SPO11, RAB3A, ITCH, LRAT, KHDRBS1, ETNPPL, TBCA, PTPRG, NRXN3, MVB12A, JAK1, SPDYA, AGO4, SESN3, PRKD1, TMEM50A, CENPC, ATP8B1, SYT11, SCN3A, TMEM68, ZNF350, RNF19B, ZNF474, PPP2R1A, AVIL, SLC35D3, ZNF257, PIK3C2B, COL2A1, VCPIP1, BAALC, CLGN, SRGAP1, TPR, LPAR4, GTF3C4, SNIP1, DCAF10, FAM117A, ZNF675, EGFR, SSH1, BCAP29, ZNF800, QSER1, MBTD1 |
